# Supplementary material for: From ADHD symptoms to parental stress: The roles of functional impairment, family functioning, and parental ADHD
Source: PLoS One. 2026 Jan 28;21(1):e0341817. doi: 10.1371/journal.pone.0341817 (PMC12851467; doi:10.1371/journal.pone.0341817)
Supplement: S1 Table — (DOCX) [file pone.0341817.s001.docx]

**S1 Table Characteristics of the included and excluded samples in this study**

|  | Included sample  (N = 127)  n (%) | Excluded sample  (N = 37)  n (%) | P value |
| --- | --- | --- | --- |
| Age, Mean (SD) | 9.6 (3.3) | 10.4 (3.2) | 0.248 |
| Sex   - Male | 89 (71.2) | 29 (78.4) | 0.398 |
| Informant   - Mother - Father - Relatives | 96 (81.4)  15 (12.7)  7 (5.9) | 26 (70.3)  8 (21.6)  3 (8.1) | 0.341 |
| Living with   - Father and Mother - Mother - Father | 66 (58.9)  34 (30.4)  4 (3.6) | 20 (55.6)  8 (22.2)  0 (0.0) | 0.053 |
| Marital status   - Married - Separated - Divorced - Widowed | 70 (63.1)  18 (16.2)  22 (19.8)  1 (0.9) | 21 (58.3)  6 (16.7)  7 (19.4)  2 (5.6) | 0.393 |
| Paternal age, Mean (SD) | 42.1 (8.4) | 43.7 (8.5) | 0.331 |
| Father’s Educational Level   - Pre-university education - Vocational Certificate /High Vocational Certificate/Diploma - Bachelor's degree - Postgraduate degree | 16 (14.2)  24 (21.2)  54 (47.8)  14 (12.4) | 5 (13.5)  8 (21.6)  14 (37.8)  5 (13.5) | 0.346 |
| Maternal age, Mean (SD) | 39.4 (6.5) | 40.9 (7.5) | 0.241 |
| Mother’s Educational Level   - Pre-university education - Vocational Certificate /High Vocational Certificate/Diploma - Bachelor's degree - Postgraduate degree | 18 (15.1)  13 (10.9)  69 (58.0)  19 (16.0) | 7 (18.9)  9 (24.3)  18 (48.6)  3 (8.1) | 0.338 |
| Family monthly income   - <15,000 baht - 15,001 - 25,000 baht - 25,001-50,000 baht - 50,001-100,000 baht - > 100,000 baht | 7 (6.3)  12 (10.8)  30 (27.0)  46 (41.4)  16 (14.4) | 1 (2.8)  3 (8.3)  14 (38.9)  15 (41.7)  3 (8.3) | 0.585 |
| ADHD DSM-5 Criteria Diagnosis   - Inattention presentation - H/I presentation - Combined presentation | 56 (44.1)  16 (12.6)  55 (43.3) | 18 (48.6)  1 (2.7)  18 (48.6) | 0.092  0.092  0.565 |
| Parental stress score, Mean (SD) | 1.8 (0.5) | 1.8 (0.5) | 0.697 |
| Child’s ADHD symptoms, Mean (SD) | 1.8 (0.6) | 1.6 (0.5)^a^ | 0.139 |
| Functional impairment score, Mean (SD) | 0.8 (0.4) | 0.8 (0.3)^b^ | 0.686 |
| Family function score, Mean (SD) | 2.4 (0.6) | 2.3 (0.4)^c^ | 0.390 |
| Parental ADHD score, Mean (SD) | 1.1 (0.8) | 1.1 (0.7)^d^ | 0.948 |

**Abbreviations :** ADHD, Attention deficit hyperactivity disorder; H/I, Hyperactivity and impulsivity

Note: *p < 0.05; **p < 0.01; ***p < 0.001

^a^ N = 7 due to missing SNAP-IV items >20%

^b^ N = 26 due to missing WFIRS-P items >20%

^c^ N = 6 due to missing SCORE-15 items >20%

^d^ N = 8 due to missing ASRS-v1.1 items >20%
