# Supplementary material for: From ADHD symptoms to parental stress: The roles of functional impairment, family functioning, and parental ADHD
Source: PLoS One. 2026 Jan 28;21(1):e0341817. doi: 10.1371/journal.pone.0341817 (PMC12851467; doi:10.1371/journal.pone.0341817)
Supplement: S5 Fig — (DOCX) [file pone.0341817.s006.docx]

**
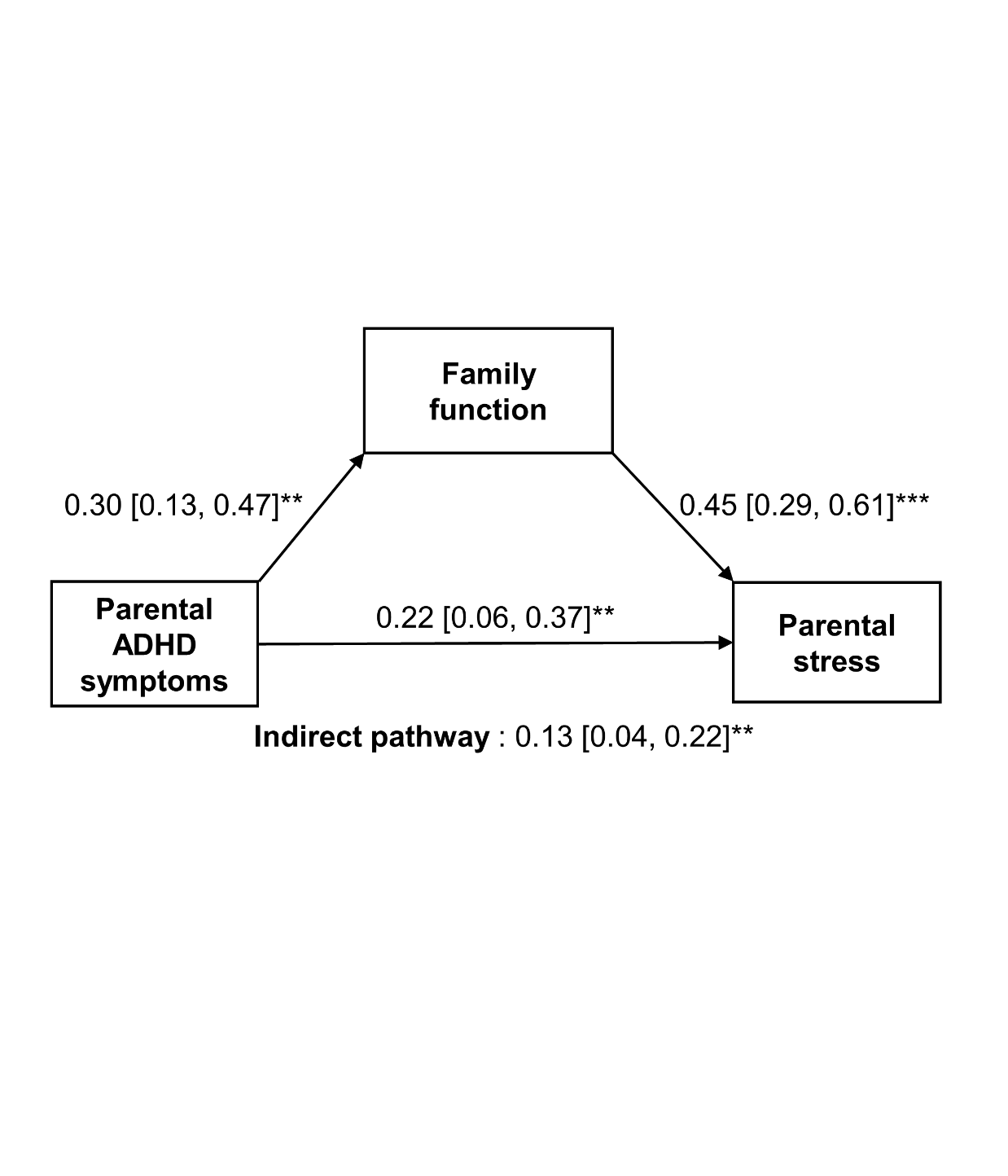
**

**S5 Fig. Sensitivity analysis additionally adjusted for comorbid specific learning disorder, assessing the paths from parental ADHD symptoms to parental stress.** The model was mediated by family function impairment and adjusted for the child’s age, sex, and comorbid specific learning disorder. Model fit indices are as follows: χ²(9) = 59.455, p < 0.001; Comparative Fit Index (CFI) = 1.000; Root Mean Square Error of Approximation (RMSEA) < 0.001; Standardized Root Mean Square Residual (SRMR) < 0.001. Significant paths are indicated by *p < 0.05, **p < 0.01, and ***p < 0.001. Sample size (N) = 117
